# A Cooperative Communication Protocol for QoS Provisioning in IEEE 802.11p/Wave Vehicular Networks

**DOI:** 10.3390/s18113622

**Published:** 2018-10-25

**Authors:** Jin-Woo Kim, Jae-Wan Kim, Dong-Keun Jeon

**Affiliations:** 1 School of Software, Soongsil University, Dongjak-gu, Seoul 06978, Korea; jjin300@ssu.ac.kr; 2 Division of Electronics & Info-Communication Engineering at Yeungjin College, Buk-gu, Daegu 41527, Korea; jwkim@yjc.ac.kr; 3 Department of Mechatronics, Incheon National University, Yeonsu-gu, Incheon 22012, Korea

**Keywords:** WAVE, cooperative communication, VANET, ITS, IEEE 802.11p, IoT

## Abstract

Vehicular ad hoc networks (VANETs) provide information and entertainment to drivers for safe and enjoyable driving. Wireless Access in Vehicular Environments (WAVE) is designed for VANETs to provide services efficiently. In particular, infotainment services are crucial to leverage market penetration and deployment costs of the WAVE standard. However, a low presence of infrastructure results in a shadow zone on the road and a link disconnection. The link disconnection is an obstacle to providing safety and infotainment services and becomes an obstacle to the deployment of the WAVE standard. In this paper, we propose a cooperative communication protocol to reduce performance degradation due to frequent link disconnection in the road environment. The proposed protocol provides contention-free data delivery by the coordination of roadside units (RSUs) and can provide the network QoS. The proposed protocol is shown to enhance throughput and delay through the simulation.

## 1. Introduction

Due to the recent rapid development of electronic and communication technologies, vehicles are expected to evolve into a vehicle ad-hoc network (VANET) that can provide drivers with safety, entertainment, and convenience. A vehicle-to-infrastructure (V2I) communication can provide commercial services such as real-time traffic information, digital maps, movies, and music through the connection with the external Internet to the vehicle. A vehicle-to-vehicle (V2V) communication can provide the driver safety information service such as collision avoidance and accident alert to the vehicle.

However, to provide various intelligent transport system (ITS) services, unlike a conventional mobile ad hoc network (MANET), it is required a vehicle network technology suitable for high speed and frequent movement of a vehicle. Various countries such as the US, Europe, and Japan are pursuing national-level projects to build infrastructures and striving to establish standards.

A dedicated short-range communication (DSRC) standard, which is widely used for road traffic information service and electronic toll collection (ETC) service, was developed to exchange information between the roadside unit (RSU) and on-board unit (OBU) in a short range. The DSRC has a transmission rate of up to 1 Mbps at a maximum speed of 160 km/h and a maximum communication range of about 100 m. However, as the ITS has been recently developed, the amount of information to be exchanged between the RSU and the OBU will increase, and a communication system with a higher transmission rate and a more extended communication range is required. Furthermore, the need for communication between the two vehicles has emerged.

The American Society for Testing and Materials (ASTM), a DSRC specification task group, has decided to explore the next generation DSRC technology to meet the requirements of the ITS while preserving the advantages of the legacy DSRC [1].

As a result of reviewing various communication technologies, the ASTM pointed out the wireless local area network (LAN) technology which is widely used in daily life and has secured stability and marketability. Based on an IEEE 802.11a standard, the ASTM defines a new specification called IEEE 802.11p that satisfies poor power supply noise and in-vehicle communication environment [2]. The service-related standard for the ITS is defined by the IEEE vehicular technology society (VTS) as IEEE P1609. The IEEE 802.11p and IEEE P1609 standards described above are collectively called a wireless access in vehicular environments (WAVE) standard [3]. In the US, 75 MHz of spectrum in the 5.9 GHz frequency band has been allocated for DSRC applications. Other frequency bands have also been used for DSRC applications even before the 5.9 GHz band allocation. Table 1 shows a spectrum allocation for WAVE/DSRC applications.

The IEEE 802.11p standard defines the MAC layer and PHY layer considering a vehicle communication environment in IEEE 802.11 which is a conventional wireless LAN standard. In the legacy IEEE 802.11 standard, an inter-node communication is possible after the completion of scanning, authentication, and association procedures. However, the IEEE 802.11p standard defines an outside context of BSS (OCB) for omitting these procedures and enabling communication. IEEE 1609 is a standard of the upper protocol layer of the WAVE standard and includes IEEE 1609.2, IEEE 1609.3, IEEE 1609.4, and IEEE 1609.12 [4,5,6,7]. IEEE 1609.12 defines the provider service identification (PSID) allocations, IEEE 1609.2 defines security service related standard, and IEEE 1609.3 defines networking related wave short message protocol (WSMP). IEEE 1609.4 also describes multi-channel operations. IEEE 1609.4 divides a wireless channel into a control channel (CCH) and a service channel (SCH) and allocates different frequency channels to each channel and switching each channel periodically.

WAVE can support a data rate up to 27 Mbps in vehicles with a speed of up to 200 km/h. In consideration of natures of a vehicle network, WAVE standard adopts a WAVE basic service set (WBSS) concept and has a different technical feature from the existing IEEE 802.11 standard [3]. The WAVE standard provides a multi-channel DSRC solution, and various services are being developed that use V2V and V2I communications based on this standard. These types of services include crash warning services, traffic information update, navigation update, and infotainment.

The WAVE communication system provides not only communication between the OBU and RSU, but also communication between two OBUs. The OBU is mounted in the vehicle and provides services to the driver and the passenger. The RSU is installed on the roadside and provides with the function of connecting to the external network. Therefore, the OBU can be connected to the external network through the RSU. Many studies related to WAVE have been performed [8,9,10,11,12,13,14,15,16,17]. However, because of the high cost for the installation of the RSU and geographical problems, the communication range of the RSUs may not include all the roads. This problem can lead to discontinuous Internet connection and disconnection to the intelligent transport system (ITS) server and can also cause severe problems for vehicle safety. The WAVE standard only supports a single-hop transmission scheme, and the RSU can transmit data frame and safety message for ITS services to vehicles in its visible range. Vehicles outside the communication range of the RSU cannot connect to the RSU and receive data frames for service applications and safety messages for ITS services. In environments where there are many shadow zones of the RSU, the loss of data frames and safety messages increases dramatically. To address these problems, a relay communication technique applicable to the VANET is required. The efficient relay decision scheme is needed to adaptively cope with the problems of link disconnection and overhead increase caused by the high mobility of the vehicle. The existing research deals with multi-hop communications in VANETs, to the best of our knowledge, schemes for multi-hop data delivery have not been proposed for the WAVE standard [18,19,20,21,22,23,24,25,26,27,28,29,30,31,32].

In this paper, we propose a cooperative communication protocol to reduce performance degradation due to frequent link disconnection in the road environment. The proposed scheme is compatible with the WAVE standard and can provide the quality of service (QoS) required by the network by reducing the delay time. The key features of our proposed protocol include the following. First, we model the path maintenance expectation time (PMET) to predict the link expiration time. Second, when a relay node is selected, a relay node with a longer PMET is selected. The proposed scheme can reduce the link disconnection between devices and improve the network performance. Lastly, since only the reserved devices communicate in the reserved resources, collision by other devices does not occur, and there is no competition for the data transmission. Thus, the proposed scheme is not subject to interference from other devices and can improve network performance. In addition, the proposed scheme is less affected by the network environment.

## 2. WAVE Protocols and Model

The WAVE PHY layer is defined in the IEEE 802.11p standard [1]. IEEE 802.11p is a modified version of the IEEE 802.11a/g standard which is a conventional wireless LAN standard. Unlike the existing wireless LAN standard, it uses the frequency of 5.850–5.925 GHz instead of industrial, scientific, and medical (ISM) band and uses 10 MHz bandwidth as one basic channel. This is to reduce the influence of frequency selective fading which occurs in high speed moving road environment by reducing channel bandwidth. While the IEEE 802.11a/g standard has to perform an authentication procedure before establishing the connection between a wireless terminal and an access point (AP), the WAVE standard does not require the authentication procedure. Therefore, a vehicle to which the WAVE standard is applied can communicate immediately if only the channel setting between the vehicles or the RSU coincides with the other.

The IEEE 802.11p standard may have security issues due to the lack of authentication procedures in existing wireless Internet standards for fast communication setup. The IEEE 1609 standard is further defined to address this problem. Currently, the IEEE 802.11p standard defines the PHY layer and MAC layer. The MAC layer and the upper layers are defined in the IEEE 1609 standard, and it is divided into four detailed definitions. The IEEE 1609.12 standard specifies allocations of WAVE identifiers defined in the IEEE 1609 series of standards. The IEEE 1609.2 standard provides security services for the WAVE networking stack and for applications that are intended to run over the stack. The IEEE 1609.3 standard specifies the functions associated with the LLC, network, and transport layers of the OSI model and calls them WAVE networking services. The IEEE 1609.4 standard provides enhancements to the IEEE 802.11p MAC to support multi-channel operations.

**Figure 1:**
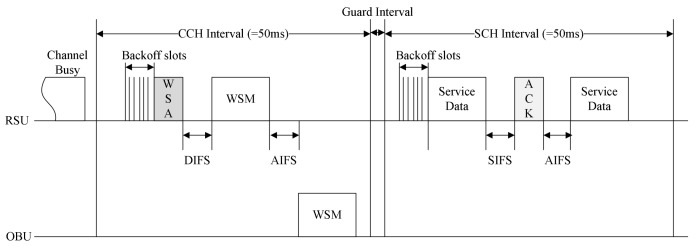
The channel structure in the WAVE standard.

The IEEE 1609.4 standard divides the wireless channel into a CCH and an SCH and allocates different frequency channels to each interval. Figure 1 shows the channel structure of the WAVE standard.

The WAVE standard uses one CCH and six SCHs. The CCH is dedicated to system control messages, and the SCH is used to exchange service data packets. The CCH interval and the SCH interval are fixed as 50 ms, respectively. The OBU receives WAVE control packets in the CCH interval and transmits or receives service data frames. The channel access in the CCH interval and the SCH interval uses the IEEE 802.11e enhanced distributed channel access (EDCA) mechanism. The WBSS, which is configured to communicate using WAVE standard, consists of a provider that starts WBSS and a user that subscribes to WBSS. The provider periodically broadcasts a WAVE service announcement (WSA) message, which is a beacon frame containing network parameters such as WBSS identifiers required to join WBSS, service channels to be used by WBSS, and timing information for synchronization. When the user receives the WSA message, it can subscribe to the WBSS by switching the channel to the service channel used by the WBSS at the next SCH interval. The CCHInterval indicates the length of the CCH interval. During this interval, the management message for the service advertisement and the data message of the high priority application are transmitted. The SCHInterval indicates the length of the SCH interval, and a general data service message is transmitted during this interval.

## 3. Related Works

In [18], the authors presented a position-based routing protocol to reduce the performance degradation by radio obstacles. The algorithm requires global information of the city topology. In [19], the authors proposed a movement-based routing algorithm for VANETs. This algorithm exploits the position and direction of movement of vehicles. The algorithm determines the routing path considering mobility related information such as speed and other movement characteristics of vehicles. In [20], the authors proposed a multi-hop routing protocol for urban area vehicular ad hoc networks without the need of any pre-installed infrastructure. The algorithm introduced a new metric called the expected disconnection degree (EDD) to estimate the quality of a route based on factors such as vehicle position, speed, and trajectory. In [21], the authors proposed a group-based routing protocol to enhance routing consistency. Vehicles are divided into four groups depending on the velocity vector. The routing protocol is considered stable if two vehicles belong to the same group. Otherwise, it is considered unsteady. In [22], the authors proposed a vehicular routing protocol to maintain routing in disconnected vehicular networks. The algorithm uses a carry-and-forward strategy to allow packets to be carried by vehicles in sparse networks for eventual forwarding when another appropriate node enters the broadcast range, thereby allowing packets to be forwarded by the relay in case of sparse networks. The VADD requires a global street map that includes traffic statistics. In [23], the authors proposed a cross-layer position-based delay-aware communication protocol called PROMPT. The PROMPT utilizes position-based source routing based on network traffic statistics collected during propagation of service advertisements of base stations. In [24], the authors proposed a diagonal-intersection-based routing (DIR) protocol for urban vehicular ad hoc networks. The algorithm is a geographic-based routing protocol. According to the geographic routing protocol, source vehicle sends data packet toward the first diagonal intersection, and then the second diagonal intersection, and so on, until toward the last diagonal intersection, and then reach to the destination vehicle. In [25], the authors proposed a unicast, multi-hop routing protocol based on opportunistic forwarding in an urban environment. The algorithm uses the information about link layer quality regarding SNIR and MAC frame error rate to improve the efficiency of the proposed routing protocol. In [26], the authors proposed a routing protocol for VANETs based on estimated network reliability. The algorithm utilizes an undirected graph representing the street layout. In this graph, the vertices are the curves or intersections in the streets and edges are street segments. In [27], the authors proposed a solution to the support of point-to-point video-streaming over VANETs. The algorithm is a receiving-based solution that uses the vehicle’s current location and their future positions estimations to better select relaying nodes. In [28], the authors proposed a multipath solution for video transmission over urban vehicular networks. The algorithm discovers relatively short paths with minimum route coupling effect based on location information. In [29], the authors proposed a contention-based forwarding protocol that dynamically selects the forwarding road segments based on their multi-hop connectivity. The algorithm selects the routing path with high probability to forward the message towards the destination. In [30], the authors proposed a geographical routing protocol to reduce the beacon overhead and to improve the routing efficiency. The algorithm forwards a packet along a street toward an intersection where the routing direction changes. In [31], the authors proposed a speed wave forecasted routing algorithm combined with speed fluctuation forecasted and computation of the movement domain to improve the GPSR greedy algorithm. The algorithm uses the vehicle speed and position to find relatively stable links, which is based on the forecast of the speed fluctuations. In [32], the authors proposed a long lifetime any paths routing protocol providing stable communication paths. The algorithm addressed the problem of stability of any path communications in vehicular ad hoc networks in the presence of inter-vehicle link failures associated with vehicle mobility. In [33], the authors proposed a MOving-ZOne-based (MoZo) architecture. The MoZo consists of multiple moving zones that group vehicles based on the movement similarity. The selected CH is responsible for managing information about CMs as well as the forwarding packets. However, these studies are routing protocols that do not conform to the WAVE standard because they do not consider the WAVE standard. In [34], the authors proposed an interference-aware relay selection to select the best relay by using inter-node interference and channel statistics. In [35], the authors investigate the issues and challenges in designing an efficient cooperative MAC scheme for multi-hop wireless networks.

In [36], the authors proposed a distributed power allocation to limit the overall interference and improve the network performance. In [37], the authors presented an analytical approach to describe the energy saving zone between a communicating pair, where a relay located inside this zone is energy efficient. Using this concept, they proposed a stochastic geometry method to estimate the energy saving gain introduced by relay-assisted D2D communication. In [38], to minimize the effect of interference at the different receivers, the authors proposed a simple opportunistic relay strategy to identify those relays providing a limited contribution to the interference. These algorithms focused on reducing interference or energy consumption. In addition, they did not consider the vehicular network environment with high mobility.

In [39], the authors proposed a dynamic-changing interval framework for the WAVE system. This scheme can shorten the transmission delay of safety messages. In [40], the authors proposed a multi-channel MAC scheme to archive high bandwidth utilization and avoid a multi-channel hidden terminal problem. In [41], the authors proposed a QoS guaranteed channel access scheme for V2V communication based on the 802.11p protocol to adjust the priority of real-time streaming to avoid collisions. However, these studies did not consider multi-hop transmission. In [42], the authors evaluated the delays and the packet delivery ratio in WAVE standard by simulation for the vehicle-to roadside link and proposed the multi-hop data delivery scheme in WAVE standard. However, this scheme did not consider the congestion by vehicle density and relay selection by the link status.

Therefore, we propose a new cooperative communication scheme that can be applied to the WAVE standard. The proposed scheme can improve the reliability and connectivity of the WAVE communication system and can provide the network QoS.

## 4. Proposed Scheme

### 4.1. Relay Node Selection

In this paper, we assume that the received signal amplitude in the vehicular networks follows the Rayleigh PDF. The Rayleigh distribution is frequently used to model multi-path fading with no direct line-of-sight (LOS) path. In vehicular networks, as the separation between source and destination devices increases, the LOS component may be lost, and the PDF of the received signal amplitude follows the Rayleigh distribution [43,44,45].

In ITS applications using a WAVE system, all WAVE devices periodically broadcast basic safety message (BSM) messages. BSM messages are messages defined in SAE J2735 to increase the safety of the vehicle in operation and frequently broadcast from all vehicles [46]. Each vehicle and RSU receives the BSM messages and determines whether it is related to the safety service. Table 2 shows the information in the BSM message.

As shown in Table 1, the BSM message includes a time (GPS time), a position of the vehicle (3D position), a position accuracy, a moving speed of the vehicle, a heading of the vehicle, the steering wheel angle of the vehicle, the acceleration of the vehicle, the break status of the vehicle, and the vehicle size. All OBUs can know the location, speed, and direction of nearby vehicles by using the received BSM message. The proposed scheme selects the relay node considering the information of the BSM message and the link status of the device requesting cooperative communication.

The existing cooperative communication schemes have selected the relay node considering the data rate on the wireless link [47,48]. However, in the VANET environment, since the moving speed of the vehicle is fast, the mobility of the vehicle greatly affects the network performance such as the packet delivery success rate. Therefore, in this paper, we calculate the path maintenance expectation time (PMET) that uses the speed and location information of the vehicle and propose the relay node selection scheme using the calculated PMET.

The OBU that does not receive the WSA can receive the BSM message from the neighboring OBUs in the CCH interval. After receiving the BSM, it calculates the PMET between the neighboring OBU and itself, the equation for the PMET is as follows.

(1) $$PMET_{i}=\frac{R-D_{i}}{2\times \left|V_{i}-V_{o}\right|},$$

where *R* is the transmission distance of OBU, and *D_i_* is the distance between itself and the *i*-th OBU. *V_i_* is the moving speed vector of the *i*-th OBU, and *V_o_* is the moving speed vector of itself.

**Figure 2:**
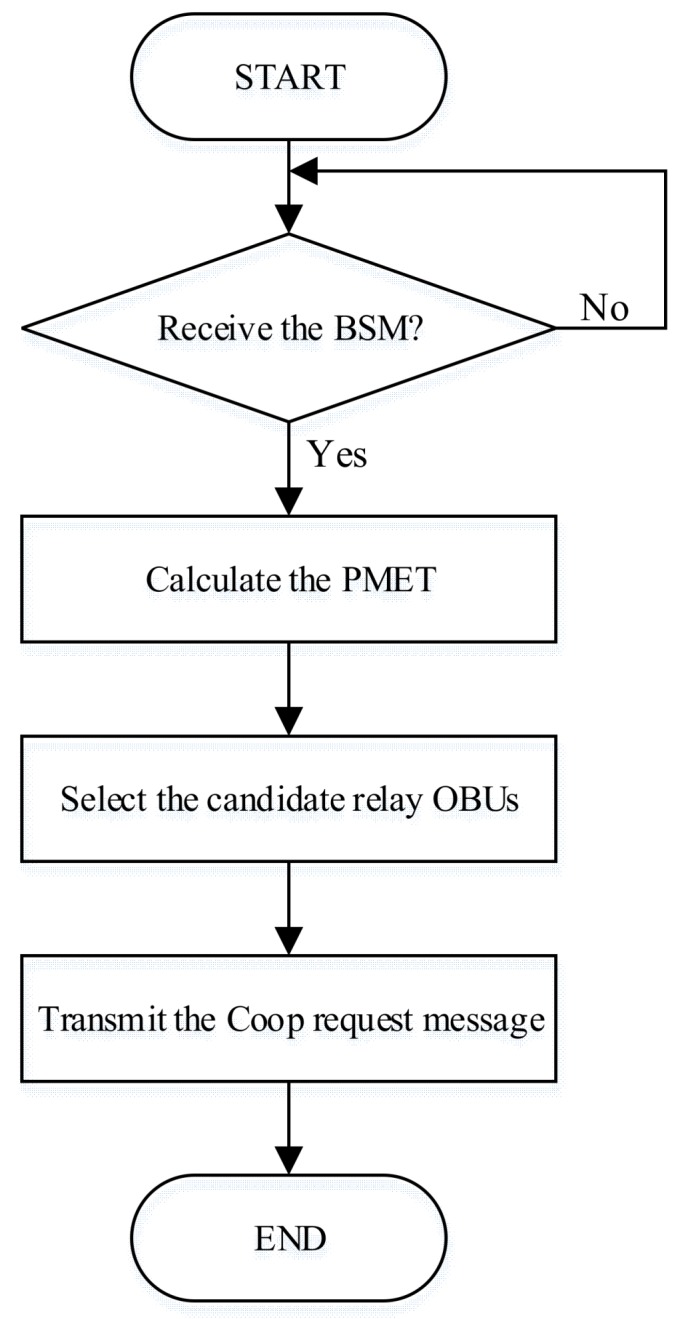
The flowchart of the on-board unit (OBU) that does not transmit a WAVE service advertisement (WSA) message.

**Figure 3:**
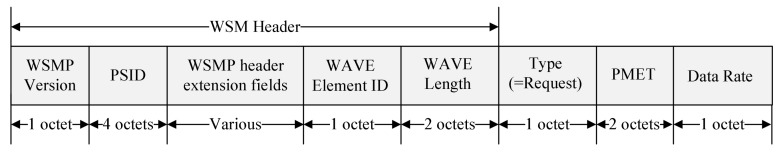
The format of the proposed Coop Request message.

After calculating the *PMET*, the OBU selects three adjacent OBUs with the longest *PMET* and transmits a Coop request message. Figure 2 shows the flowchart of the OBU that does not receive the WSA, and Figure 3 shows the structure of the proposed Coop Request message.

**Figure 4:**
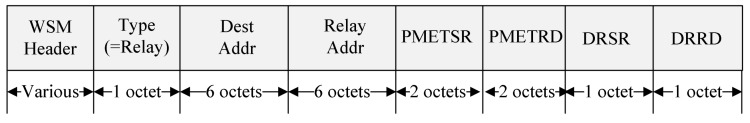
The format of the proposed Coop Relay message.

WSMP Version field shows the version of the WAVE protocol, PSID (Provider Service Identifier) is a numerical field used by the IEEE1609 standard to identify a particular application. To get access to the WAVE service, an application should be registered with its unique PSID. The WAVE provider devices use PSID in its announcement messages to indicate that it provides a certain application. WSMP header extension field defines the channel that is used for communication. The PMET field indicates the calculated PMET value. The Data Rate field is set to the data rate that the recipient device recommends to the source device. OBUs receiving the Coop Request message transmit a Coop Relay message to the RSU, and Figure 4 shows the format of the proposed Coop Relay message.

The Type field indicates whether the transmitted message is a request or a relay. The PMETSR field indicates the PMET between the OBU requesting the cooperative communication and the relay candidate OBU, and the PMETRD field indicates the PMET between the relay candidate OBU and the RSU. The DRSR field indicates the data rate between the OBU requesting the cooperative communication and the relay candidate OBU, and the DRRD field indicates the data rate between the relay candidate OBU and the RSU.

RSU receiving Coop Relay message has to calculate the total transmission time to transmit WSA message from source to destination. Considering the Rayleigh fading model, the received SNR has exponential distribution given by [49]:

(2) $$f\left(\gamma \right)=\frac{1}{\bar{\gamma }}\exp\left(-\gamma /\bar{\gamma }\right),\;\gamma \geq 0$$

where $\bar{\gamma }$ is the average SNR. The probability that the data frame is correctly received at a distance d is given by

(3) $$P\left[\gamma \left(d\right)\geq \psi \right]=\exp\left(-\psi /\bar{\gamma }\right)=\exp\left[-d^{\alpha }W\psi /P_{tx}K\right]$$

where *P_tx_* is the transmit power, a is the path loss exponent and *K* is a constant associated with the path loss model. *K* has given by [49,50,51]:

(4) $$K=\frac{G_{T}G_{R}C^{2}}{{\left(4\pi f_{c}\right)}^{2}}$$

where *G_T_* and *G_R_*, respectively, represent the transmit and receive antenna gains. *C* is the speed of light, and *f_c_* is the carrier frequency. In this paper, we assume that the antennas are omni-directional (*G_T_* = *G_R_* = 1), and the carrier frequency *f_c_* = 5.9 GHz. Let *R_x_* denote *x* Mbps data rate. The probability that rate *R_x_* is achievable is calculated as follows:

(5) $$\begin{array}{l} P\left(r=R_{3}\right)=P\left(\gamma \left(l\right)<\psi_{4.5}\right)\\ P\left(r=R_{4.5}\right)=P\left(\psi_{4.5}\leq \gamma \left(l\right)<\psi_{6}\right)\\ P\left(r=R_{6}\right)=P\left(\psi_{6}\leq \gamma \left(l\right)<\psi_{9}\right)\\ P\left(r=R_{9}\right)=P\left(\psi_{9}\leq \gamma \left(l\right)<\psi_{12}\right)\\ P\left(r=R_{12}\right)=P\left(\psi_{12}\leq \gamma \left(l\right)<\psi_{18}\right)\\ P\left(r=R_{18}\right)=P\left(\psi_{18}\leq \gamma \left(l\right)<\psi_{24}\right)\\ P\left(r=R_{24}\right)=P\left(\psi_{24}\leq \gamma \left(l\right)<\psi_{27}\right)\\ P\left(r=R_{27}\right)=P\left(\gamma \left(l\right)\geq \psi_{27}\right) \end{array}$$

where *ψ_x_* is the minimum required SNR threshold to support x Mbps data rates.

RSU receiving Coop Relay message has to calculate the total transmission time to transmit WSA message from source to destination. Their transmission times are calculated as follows:

(6) $$T_{CT}=P\left(r=R_{SR}\right)\cdot \frac{8L_{W}}{R_{SR}}+P\left(r=R_{RD}\right)\cdot \frac{8L_{W}}{R_{RD}}+2\cdot T_{DISF},$$

where *L_W_* is the size of WSA message. *R_SR_* and *R_RD_* are the data rate from source to relay, and from relay and destination. The *T_DIFS_* is the interframe space interval defined by the IEEE 802.11p specification.

The *RSU* calculates the relay decision parameter (*RDP*) of the relay candidates and selects the OBU with the lowest *RDP* as the relay node. The *RDP* is calculated as follows.

(7) $$RDP=\frac{T_{CT}}{PMET_{SR}+PMET_{RD}}$$

### 4.2. Resource Allocation Scheme for Cooperative Communication

**Figure 5:**
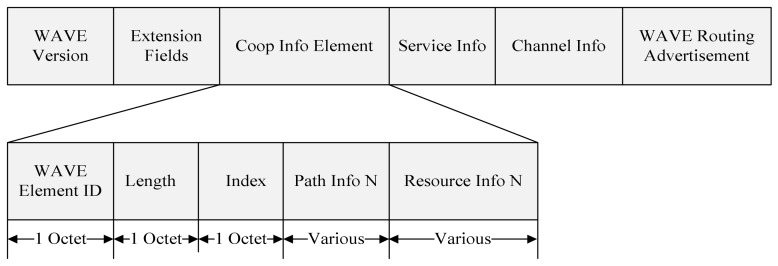
The format of the proposed WSA message.

Figure 5 shows the format of a proposed WAVE service advertisement (WSA) message.

The RSU announces the type of service applications to OBUs using the Service Info field. Using the Channel Info field, the RSU also announces the number of the channel in which it provides the service. The WAVE Routing Advertisement element provides information about infrastructure internetwork connectivity, allowing receiving devices to be configured to participate on the advertised IPv6 network.

**Figure 6:**
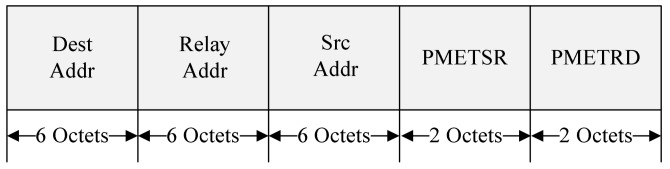
The format of the proposed Path Info field.

The Coop Info Element includes a WAVE Element ID, Length, Index, Path Info, and Resource Info fields. The value of the WAVE Element ID is selected one of the reserved values so that the proposed Coop Info Element can be distinguished. The Length field indicates the length in octets of Index, Path Info, Resource Info fields. The index field contains the number of relay paths included in the Coop Info Element. Figure 6 shows the format of the proposed Path Info field.

**Figure 7:**
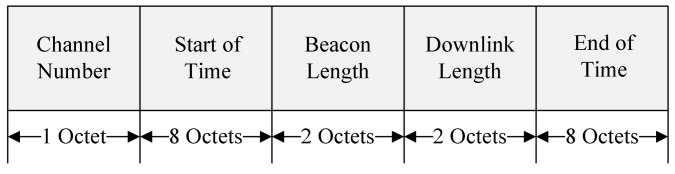
The format of the proposed Resource Info field.

The Dest Addr field indicates the MAC address of the device requesting the cooperative communication and the Relay Addr field contains the MAC address of the OBU selected as the relay node. The Src Addr field is set to the MAC address of the RSU. The PMETSR field indicates the PMET between the RSU and the relay node, and the PMETRD field indicates the PMET between the relay node and the destination node. Figure 7 shows the format of the proposed Resource Info field.

**Figure 8:**
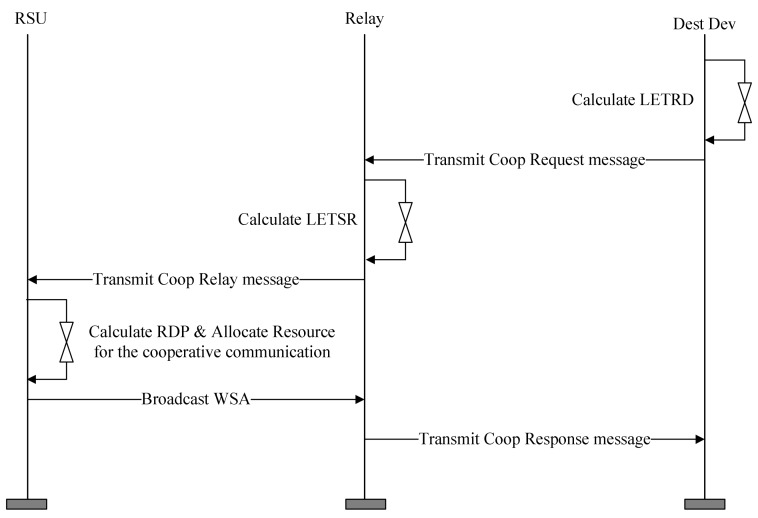
The timing diagram of the path construction and the resource allocation for the cooperative communication.

The Channel Number field indicates the SCH channel selected by the RSU for relay communication. The Start of Time field indicates the beginning of the resource allocated for the relay, which specifies the universal coordinated time (UTC) second of GPS. The Beacon Length field is the interval to relay the WSA message to the destination node. The Upper Link Length field is the interval that the RSU has allocated to communicate with the relay node. The remaining allocated resources are the intervals allocated for the relay node and the destination node to communicate. The End of Time field indicates the end of the resource allocated for relay communication and specifies the UTC second. Figure 8 shows the timing diagram of the path construction and the resource allocation for the cooperative communication.

**Figure 9:**
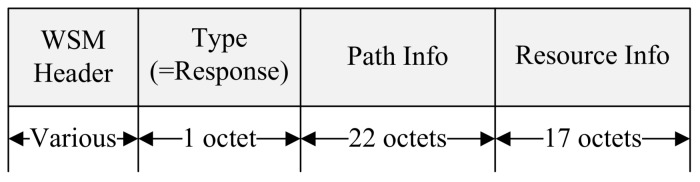
The format of the proposed Coop Response message.

The RSU that receives the Coop Relay message from the relay candidate nodes calculates the RDP using the information in the received message and selects the OBU with the lowest RDP as the relay node. The RSU allocates resources for the cooperative communication in the SCH Interval and broadcasts the WSA including the information on the relay path and the allocated resources. The relay node receiving the WSA message generates a Coop Response message using the information included in the WSA. Figure 9 shows the format of the proposed Coop Response message.

**Figure 10:**
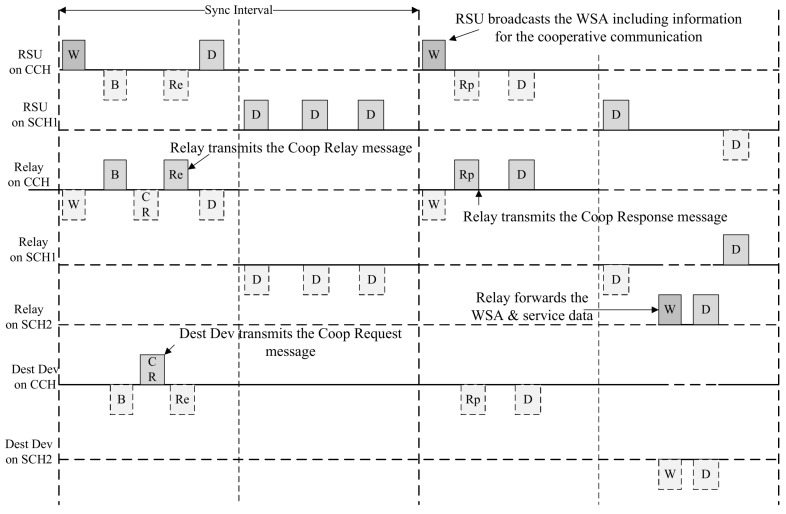
An example for the proposed cooperative communication scheme.

The relay node sets the Type field Response and sets the Path Info and Resource Info fields to the values of the Path Info and Resource Info fields contained in the received WSA. The relay node transmits the Coop Response message to the destination device. The OBU receiving the Coop Response message checks the relay node selected from the Path Info field and ascertains the SCH channel and the allocated resource to perform cooperative communication from the Resource Info field. The relay node forwards the received WSA message to the destination node. Devices on the relay path exchange data frames in the allocated Relay interval. Figure 10 shows an example of the proposed cooperative communication scheme.

**Figure 11:**
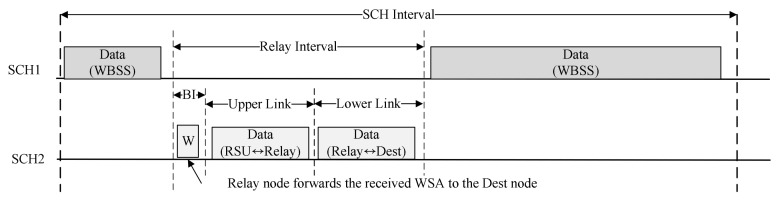
An example of data exchange in service channel (SCH) interval.

In Figure 10, the Dest Dev computes the PMET after receiving the BSM message from the neighboring OBU. It transmits a Coop Request message including the calculated PMET and the data rate supported by the link to the neighboring OBU. The OBU receiving the Coop Request message calculates the PMET with the RSU and transmits a Coop Response message including the calculated PMET and the data rate to the RSU. The RSU receiving the Coop Response message calculates the RDP based on the received link information and selects the relay node based on the calculated RDP. RSU also selects the SCH Interval for cooperative communication and broadcasts the WSA message including information for the relay path and the allocated SCH interval. The relay node receiving the WSA ascertains the relay path information, and sends the Coop Response message including the received information back to the Dest Dev. The Dest Dev receiving the Coop Response message checks the information for the cooperative communication and requests a service to the RSU in the allocated SCH interval. Figure 11 shows an example of exchanging data between the RSU and the destination node in the SCH interval.

In Figure 11, devices participating in WBSS perform communication in SCH 1 channel. During the Relay Interval allocated through the Coop Response message, the RSU and the relay node change their channels to the SCH 2 channel and exchange the WSA message and data frames with the destination device. During the Upper Link interval, the RSU and the Relay node communicate with each other, and during the Lower Link interval, the relay node and the destination node communicate with each other. After the Relay interval, the RSU and the relay node return to the SCH 1 channel and communicate with OBUs in the WBSS. Since all devices in the WBSS are aware of the information of the Relay Interval through the Coop Response message, they do not transmit data frames to the RSU and the relay node in the Relay interval.

### 4.3. Release of the Resource Reserved for the Cooperative Communication

**Figure 12:**
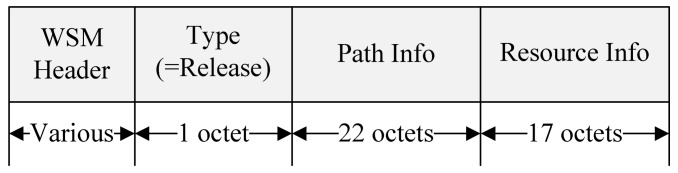
The format of the proposed Coop Release message.

If the relay node does not receive data or response from the destination node for a certain period, it performs reservation resource release procedure. If cooperation communication is no longer needed, the destination node also performs a resource reservation release procedure. To release the reserved resource, the destination node sends a Coop Release message to the relay node. Figure 12 shows the format of the proposed Coop Release message.

**Figure 13:**
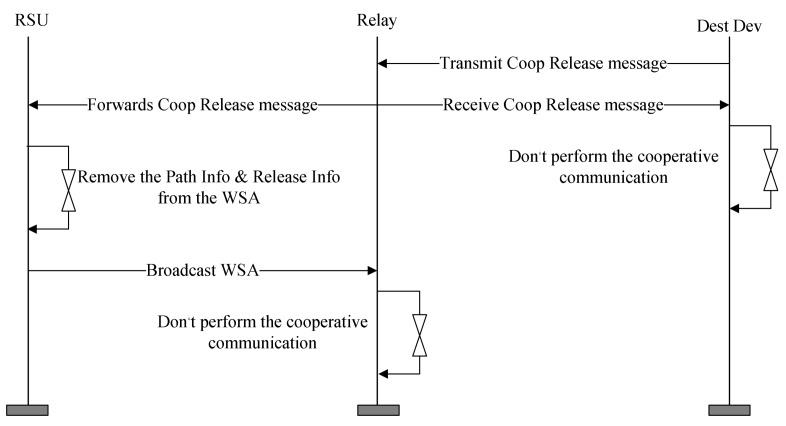
The timing diagram for the exchange of the control message for the resource release.

To terminate the relay communication, the relay node sets the Type field to Release and includes the information of the routing path and resources used for the cooperative communication to the Coop Release message. When the relay node receives the Coop Release message from the destination node, it forwards the Coop Release message to the RSU. After receiving the Coop Release message, the RSU broadcasts the WSA message except for the corresponding information at the next Sync Interval. The relay node that receives the WSA message no longer participates in the cooperative communication, and the destination node that receives the Coop Release message also no longer performs the cooperative communication. Figure 13 shows the timing diagram for the exchange of the control message for the resource release.

## 5. Performance Evaluation

For evaluation purposes, we compare our proposed scheme with the WAVE standard and overhearing-driven counter-based delayed forwarding (OCBDF) scheme in WAVE standard [42]. Performance of the proposed scheme and other protocols are evaluated using the discrete event simulator Omnet++ [52]. The simulation parameters are summarized in Table 3.

A radio module implements the 802.11p PHY and MAC model of OMNET++, and parameters are set according to the recommended values in [1]. We consider a Rayleigh fading model with the path-loss exponent of 2.5. RSUs are uniformly distributed along the road segment with a predefined distance. Simulations are run for a duration of 600 s, and we limit the communication path to 2-hop. 100 vehicles move with speed ranging from 35 to 100 km/h. In this simulation, we consider 10 km highway. RSUs are deployed uniformly in the highway. In this highway, the vehicles can move in two opposite directions, and there are three lanes in each direction of the highway.

**Figure 14:**
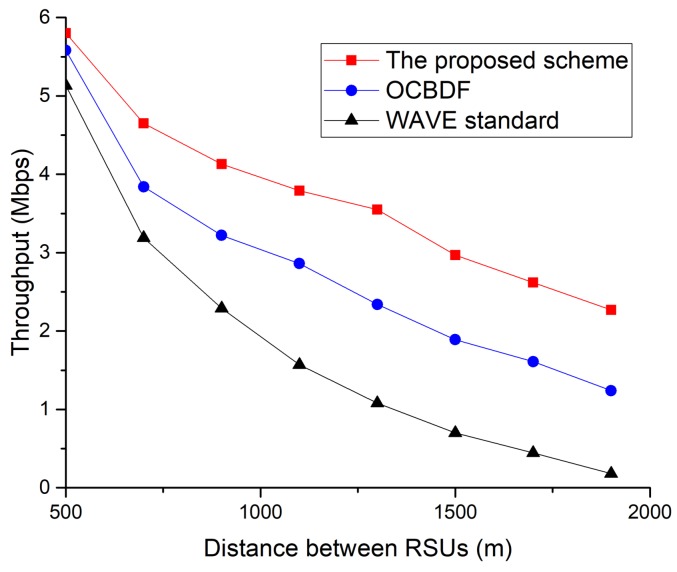
The throughput as a function of the distance between road-side units (RSUs).

Figure 14 shows the throughput obtained when the distance between RSUs increases.

**Figure 15:**
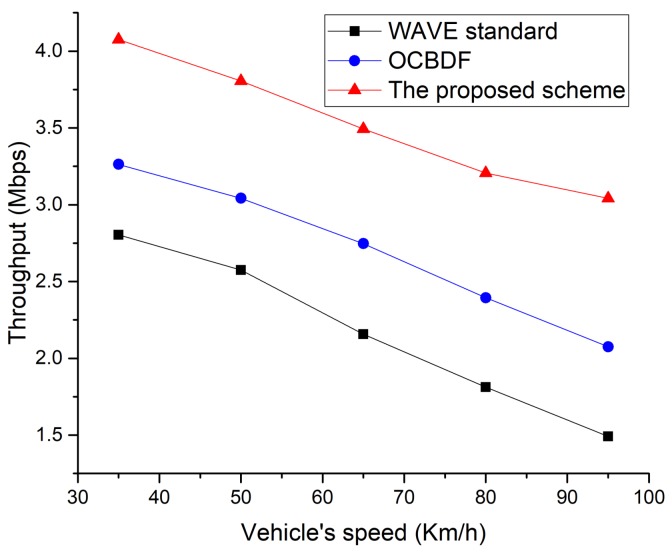
Throughput as a function of the vehicle’s speed.

As shown in Figure 14, the performance of the proposed scheme outperforms the legacy schemes, and it is also observed that the throughput drops for all schemes. This is because the shadow zone exists between RSUs. Although the distance between RSUs increases, the proposed scheme and OCBDF scheme is likely to find a two-hop connection to an RSU. However, the performance of the OCBDF scheme is getting worse since the OCBDF scheme does not consider the link status to select the relay node. Figure 15 shows the throughput as a function of the vehicle’s speed.

**Figure 16:**
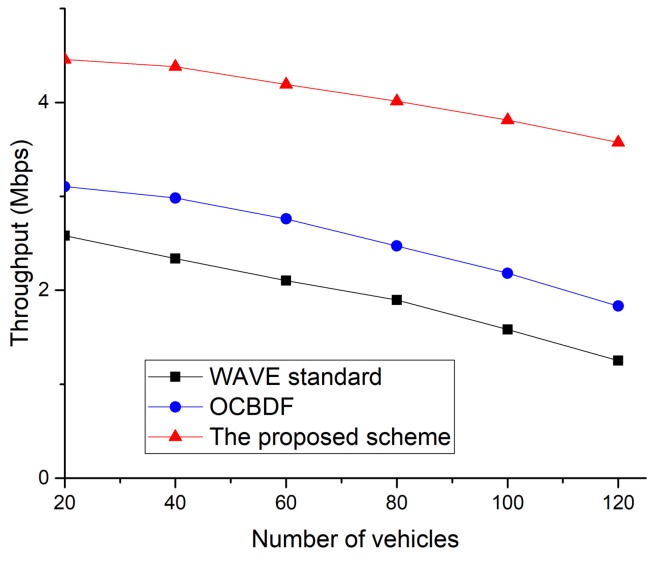
Throughput as a function of the number of vehicles.

In this simulation, we fixed the distance between RSUs to 1000 m. In Figure 14, it is observed that the performance of the WAVE standard is worst. This is due to the existence of the shadow zone between the adjacent RSUs. The performance of the OCBDF scheme is superior to the WAVE standard since the OCBDF scheme can transmit data frames through the two-hop connection. However, when the speed of vehicles increases, link disconnection frequently occurs, and the throughput of the OBCDF scheme decreases. The proposed scheme performs better than two existing schemes since it selects the relay node considering the speed of vehicle and link status. However, the proposed scheme also cannot avoid link disconnection resulting from the increase of vehicle speed, and the performance of the proposed scheme is also degraded. Figure 16 shows the throughput as a function of the number of vehicles.

**Figure 17:**
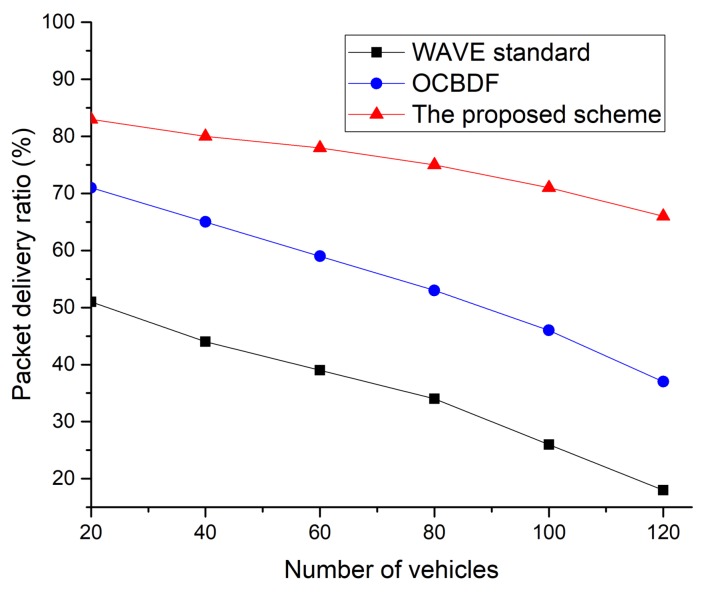
Packet delivery ratio as a function of number of vehicles.

In Figure 16, the trends of throughput regarding the number of vehicles on the road can be observed. For both the WAVE standard and OCBDF scheme, the throughput decreases when the number of vehicles on the road increases. This is the result of an increase in congestion when there are more nodes in the vehicular network. In addition, when the number of vehicles increases, the LOS between vehicles is frequently lost, and the packet loss also increases. In the proposed scheme, because OBUs can transmit data frames without the contention in the allocated SCH interval, the proposed scheme can provide better throughput than the legacy protocols. Figure 17 shows the packet delivery ratio as a function of the number of vehicles.

**Figure 18:**
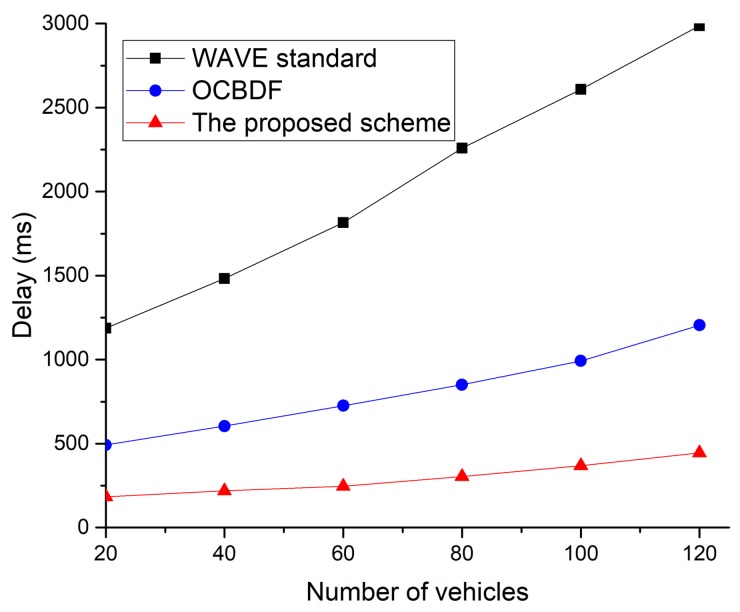
Delay as a function of the number of vehicles.

In Figure 17, the WAVE standard archives the lowest packet delivery ratio. This is because the WAVE standard is affected by the shadow zone between neighboring RSUs and the congestion. The OCBDF scheme is less affected by the shadow zone between neighboring RSUs, but it cannot avoid the congestion due to an increase in the number of vehicles. However, the proposed scheme is less affected by the shadow zone between neighboring RSUs and the congestion since OBUs can transmit data frames using the two-hop connection and can transmit data frame without contention. Therefore, the proposed scheme shows better packet delivery ratio than the legacy two protocols. Figure 18 shows the delay as a function of the number of vehicles.

In Figure 18, the proposed scheme archives the lowest delay of the three protocols. This is because the proposed scheme can transmit data frames without contention in the allocated SCH interval. When the number of vehicles increases in the vehicular network, the congestion in the WAVE standard increases. The WAVE standard does not support the multi-hop communication. In the shadow zone between neighboring RSUs, the link disconnection frequently occurs, and the OBUs cannot receive service data frames from RSUs. Therefore, the delay in packet delivery is significantly increased. The OCBDF scheme is less affected by link disconnection, but the delay of the OCBDF scheme increases due to the congestion.

## 6. Conclusions

In this paper, we have proposed a cooperative communication protocol to improve the link connectivity in the WAVE standard. In particular, we have studied the limitations in the WAVE standard for the congestion and the shadow zone between neighboring RSUs and have proposed the cooperative communication scheme to address those limitations. The proposed scheme has been demonstrated to notably improve the network performance even when a low presence of infrastructure results in shadow zones between areas of coverage. The proposed scheme also has been designed according to the intricacies and special characteristics of the WAVE standard.

The proposed scheme provides better performance than the existing algorithm. However, when service applications provided by the RSU increase, the number of SCHs for service applications increases, and the available resources for the relay communication are also reduced. As service applications provided to automobiles increase, the use of the proposed algorithm becomes more difficult. However, since the proposed algorithm can transfer data without the contention in reserved resources, collisions by access of multiple OBUs do not occur. Therefore, the proposed algorithm can provide a high throughput, a high delivery success ratio, and a low delay regardless of the number or speed of the vehicle. In the future, we will introduce cognitive radio algorithms and develop a relay communication protocol that operates in an environment where various service applications are provided. We will also develop OBU and RSU to perform field tests for the proposed algorithm.

Our simulation results show that the WAVE standard may extend the area of coverage using multi-hop communications, and the proposed scheme can reduce performance degradation due to frequent link disconnection caused by frequent vehicle speed changes. The proposed scheme can also provide the QoS since OBUs can transmit data frames without the contention. In the proposed scheme, the RSU can choose the best relay based on different parameters, such as relay reliability and link duration.

## Figures and Tables

**Table 1:** Spectrum allocation for wireless access in vehicular environments (WAVE)/dedicated short-range communication (DSRC) applications.

Country/Region	Frequency Band
ITU-R (ISM band)	5725–5875
Europe	5795–5815, 5855/58755905/5925
North America	902–928, 5850–5925
Japan	715–725, 5770–5850

**Table 2:** Basic safety message (BSM) information.

Type	Description	Size (byte)
DSRCmsgID	Data elements used in each message to define the Message type	1
MsgCount	It can check the flow of consecutive messages having the same DSRCmsgID received from the same message sender.	1
TemporaryID	Represents a 4-byte temporary device identifier. When used in a mobile OBU device, this value is periodically changed to ensure anonymity.	4
Dsecond	Represents two bytes of time information.	2
Latitude	Represents the geographic latitude of an object.	4
Longitude	Represents the geographic longitude of an object.	4
Elevation	Represents an altitude measured by the WGS84 coordinate system.	2
PositionAccuracy	Various quality parameters used to model the positioning accuracy for each given axis.	4
TransmissionAndSpeed	Represents the speed of the vehicle.	2
Heading	The current direction value is expressed in units of 0.0125 degrees.	2
SteeringWheelAngle	Represents the current steering angle of the steering wheel.	1
AccelerationSet4Way	It consists of three orthogonal directions of acceleration and yaw rate.	7
BrakeSystemStatus	Represents a data element that records various control states related to braking of the vehicle.	2
VehicleSize	Represents the length and width of the vehicle.	3

**Table 3:** Simulation Parameters.

Parameters	Value
Frequency band	5.8 GHz
Physical/MAC Layer	IEEE 802.11p
Tx Power RSU	50 mW (500 m radio range)
Tx Power OBU	11 mW (250 m radio range)
Distance between neighboring RSUs	500 m ~ 2000 m
Average speed	35 Km/h (default) ~ 100 Km/h
Simulation Time	600 s
Data Packet Length	512 Byte
